# Epstein–Barr Virus and Multiple Sclerosis: Mechanistic Insights into Virus-Driven Autoimmunity

**DOI:** 10.3390/microorganisms14081639

**Published:** 2026-07-27

**Authors:** Stavros Bashiardes, George Krashias, Elissa Englezou, Anastasia Lambrianides, Giorgos Pitsas, Marios Pantzaris, Jan Richter

**Affiliations:** 1 Postgraduate School, Cyprus Institute of Neurology and Genetics, Nicosia 2371, Cyprus; sbash@cing.ac.cy (S.B.); georgek@cing.ac.cy (G.K.); elissae@cing.ac.cy (E.E.); nancyl@cing.ac.cy (A.L.); pantzari@cing.ac.cy (M.P.); 2 Department of Molecular Virology, Cyprus Institute of Neurology and Genetics, Nicosia 2371, Cyprus; 3 Department of Neuroimmunology, Cyprus Institute of Neurology and Genetics, Nicosia 2371, Cyprus; 4 Neurology Clinic, Cyprus Institute of Neurology and Genetics, Nicosia 2371, Cyprus; giorgosp@cing.ac.cy

**Keywords:** multiple sclerosis, Epstein–Barr virus, EBNA1, antibody, molecular mimicry, CNS

## Abstract

Multiple sclerosis (MS) is a chronic inflammatory disease of the central nervous system characterized by immune-mediated demyelination and neurodegeneration. Although the exact cause of MS remains unclear, accumulating epidemiological and immunological evidence strongly implicates Epstein–Barr virus (EBV) infection as a major environmental factor associated with disease development. Nearly all individuals with MS are EBV seropositive, and longitudinal studies have demonstrated that EBV infection precedes MS onset, supporting a causal relationship. EBV establishes lifelong latency in B cells and can profoundly influence host immune responses, providing several potential mechanisms through which it may contribute to autoimmunity. In this review, we summarize current knowledge of EBV biology and discuss epidemiological findings linking EBV infection with MS risk. We then examine alterations in EBV-specific immune responses observed in MS, including dysregulated humoral and cellular immunity. Particular attention is given to molecular mimicry involving the Epstein–Barr nuclear antigen 1 (EBNA1) and central nervous system proteins, which may promote cross-reactive autoimmune responses. Finally, we discuss evidence for the presence and potential role of EBV-infected immune cells within the MS brain and highlight key unanswered questions that remain critical for understanding EBV-driven neuroinflammation.

## 1. Introduction

Multiple sclerosis (MS) is a chronic inflammatory disease of the central nervous system characterized by immune-mediated demyelination and progressive neuroaxonal degeneration. The disease typically affects young adults and represents one of the leading causes of non-traumatic neurological disability in this population [1]. Pathologically, MS is marked by focal inflammatory lesions within the brain and spinal cord, accompanied by myelin loss, axonal injury, and gliosis [2]. Although the precise cause of MS remains incompletely understood, it is widely accepted that disease development results from a complex interaction between genetic susceptibility and environmental factors that trigger dysregulated immune responses against central nervous system antigens [3,4,5]. In recent years, increasing attention has focused on the potential role of viral infections as environmental contributors to MS pathogenesis. Among environmental factors implicated in MS, Epstein–Barr virus (EBV) is considered the strongest candidate because EB infection precedes disease onset and nearly all MS patients are EBV seropositive [6]. Understanding the mechanisms through which EBV infection may contribute to MS development has therefore become a major focus of current research.

Despite extensive epidemiological evidence linking EBV infection to the development of MS, the biological mechanisms underlying this association remain incompletely understood. EBV possesses several characteristics that may contribute to autoimmune pathology, including its ability to establish lifelong latency in B cells and to modulate host immune responses. In this review, we first summarize key aspects of EBV biology and latency and discuss the epidemiological data that associate EBV infection with MS. We then examine alterations in EBV-specific immune responses observed in MS, including humoral and cellular immune responses. Particular attention is given to proposed autoimmune mechanisms such as molecular mimicry between EBV antigens and central nervous system (CNS) proteins. Finally, we discuss the potential role of EBV-infected immune cells in CNS inflammation and highlight areas where further research is needed to clarify the contribution of EBV to MS pathogenesis.

## 2. EBV Biology and Latency in B Cells

EBV, also known as human herpesvirus 4 (HHV-4), is a 172-kb double-stranded DNA γ-herpesvirus and was the first virus identified to cause human cancer [7]. Initially discovered as the causative agent for Burkitt lymphoma, EBV has since been associated with several malignancies, including Hodgkin lymphoma, non-Hodgkin lymphoma in immunocompromised individuals, T-cell and NK/T-cell lymphomas, and nasopharyngeal carcinoma [8,9]. In addition to malignancies, EBV has also been linked to infectious mononucleosis (IM), oral hairy leukoplakia, and autoimmune diseases such as systemic lupus erythematosus [10].

EBV is primarily transmitted through saliva and initially infects epithelial cells of the oropharynx during primary infection. The virus subsequently infects B lymphocytes within the tonsillar lymphoid tissue and subsequently enters the blood circulation, where it establishes lifelong persistence within the memory B-cell compartment, the principal reservoir of the virus [11,12]. Infection of B cells and epithelial cells is governed by different receptors and entry mechanism for each. In B cells, viral attachment is initiated when the EBV glycoprotein gp350 binds to the complement receptor CD21 (CR2) on the B-cell surface [13,14]. After attachment, the viral glycoprotein gp42, together with the gH/gL complex, interacts with HLA class II molecules. This interaction activates the viral fusogen gB, which mediates membrane fusion and allows the virus to enter the B cell [12]. In contrast, EBV infection of epithelial cells occurs through a more complex mechanism. Epithelial cells typically do not express CD21 or HLA class II molecules, so EBV relies on alternative receptors such as EPHA2, neuropilin-1, integrins, and non-muscle myosin heavy chain IIA. In these cells, viral glycoproteins gH/gL and gB are also required for membrane fusion, but gp42 actually inhibits infection, unlike in B cells where it promotes entry [15]. Recent evidence suggests that R9AP may function as a shared receptor that interacts with the gH/gL complex and facilitates EBV entry into both epithelial cells and B cells. Through this interaction, gH/gL activates gB-mediated membrane fusion, enabling viral uptake [16]. This finding suggests that EBV entry into different cell types may involve both cell-specific receptors and shared fusion mechanisms.

**Figure 1:**
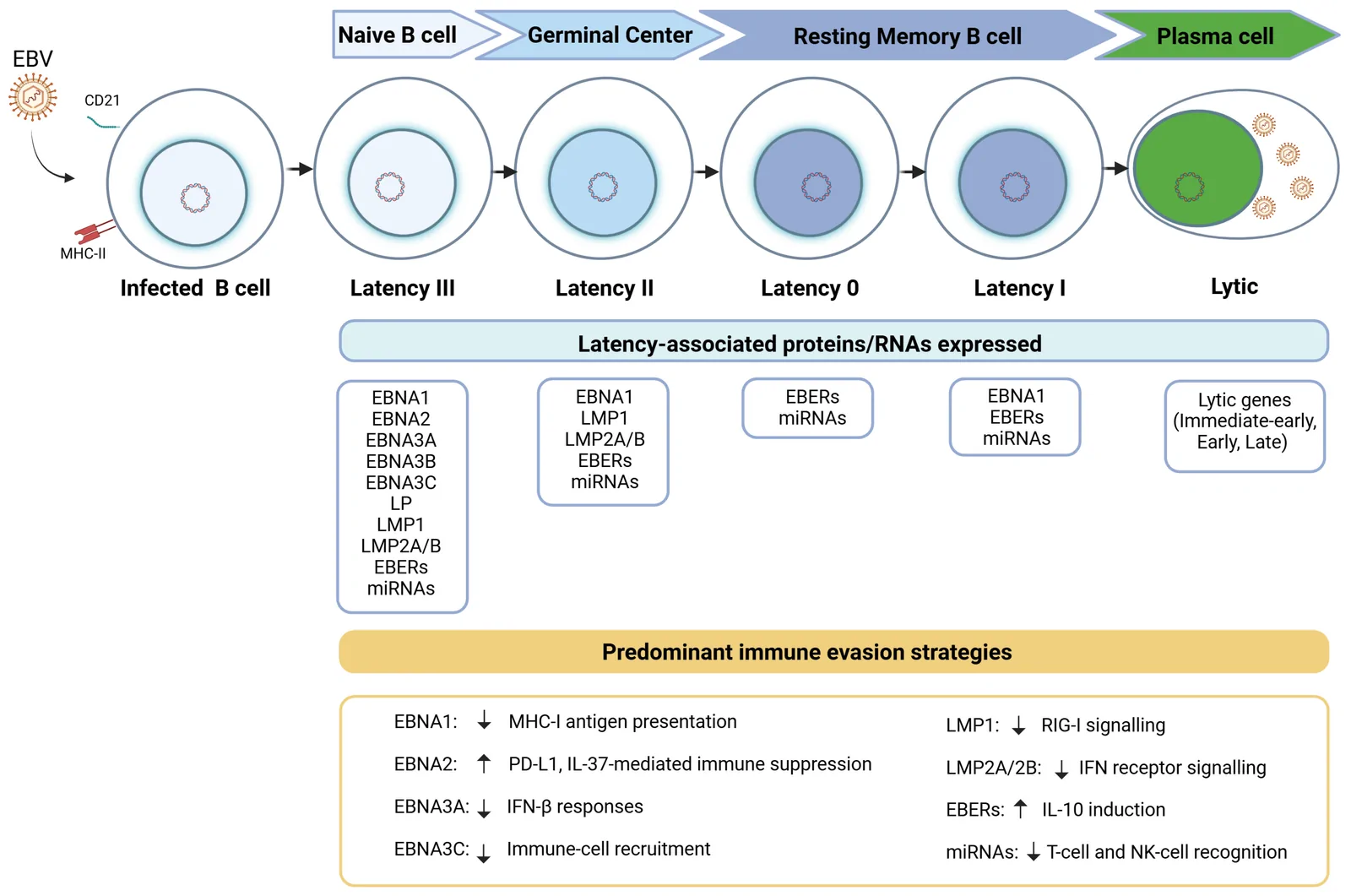
**EBV latency programs and the expression of latency-associated viral proteins, non-coding RNAs, and immune evasion mechanisms.** EBV enters naïve B cells through interaction of viral glycoproteins with the B-cell receptor CD21 and MHC class II molecules. Following infection, the virus initially drives proliferation of infected B cells under the latency III program, in which multiple latent proteins are expressed. As these cells participate in germinal center reactions, viral gene expression becomes more restricted (latency II), supporting survival and differentiation of infected B cells. In long-lived resting memory B cells, EBV persists in a largely transcriptionally silent state (latency 0), with occasional expression of EBNA1 during cell division (latency I) to maintain the viral genome. Upon activation of infected B cells and their differentiation into plasma cells, immediate-early viral genes initiate the lytic cycle, leading to viral replication and production of new virions. The figure also summarizes the principal latency-associated viral proteins and non-coding RNAs expressed during each stage of latency, together with their representative immune evasion mechanisms that facilitate viral persistence within the host. ↑: increase in abundance; ↓: decrease in abundance.

EBV maintains lifelong infection in B cells by regulating viral gene expression according to the differentiation state of the host cell. As infected B cells progress through the germinal center reaction and differentiate into memory B cells, the virus transitions through distinct latency programs that enable long-term persistence within the B-cell compartment (Figure 1). After infecting naïve B cells, the virus first induces a highly active transcriptional program known as latency III, in which all latent genes are expressed, including EBNA1–3 and the latent membrane proteins LMP1 and LMP2A/B, under the control of the transcription factor EBNA2. This program drives B-cell activation and proliferation [17,18]. As infected cells enter the germinal center, EBV adopts a more restricted latency II pattern, characterized by expression of EBNA1, LMP1, and LMP2A. During this stage, LMP1 mimics CD40 signaling and activates NF-κB pathways, promoting cell survival, whereas LMP2A provides signals similar to B-cell receptor activation, supporting continued B-cell differentiation. Once infected cells differentiate into long-lived memory B cells, viral gene expression becomes highly limited [19]. In latency 0, only non-coding RNAs such as EBERs and BART microRNAs are produced, allowing infected cells to evade immune detection [20]. During memory B-cell division, EBNA1 may be transiently expressed (latency I) to ensure replication of the viral episome. Under certain conditions, particularly during plasma cell differentiation, latent infection can switch to the lytic cycle, in which immediate-early genes (BZLF1 and BRLF1), early genes involved in DNA replication, and late genes encoding structural proteins are sequentially expressed, ultimately leading to the production of new infectious virions [19].

## 3. Epidemiological Evidence Linking EBV to MS

A strong epidemiological association between EBV infection and MS has been recognized for several decades. Early seroepidemiological studies demonstrated that nearly all individuals with MS are seropositive for EBV, whereas MS rarely occurs in EBV-seronegative individuals, suggesting that EBV exposure may be a prerequisite for disease development [21,22,23]. In addition, a history of IM, which reflects symptomatic primary EBV infection, has consistently been associated with a significantly increased risk of developing MS later in life [24,25,26,27,28,29,30,31,32]. Further support for this link comes from studies showing elevated antibody responses against EBV antigens, particularly EBNA1 [32,33,34,35,36,37], years before the onset of clinical disease [38]. More recently, large prospective cohort studies have strengthened the evidence for a causal relationship by demonstrating that EBV infection precedes MS onset. In a seminal longitudinal study, Bjornevik and colleagues analyzed serial serum samples collected from more than 10 million U.S. military personnel over a 20-year period and demonstrated that EBV infection consistently preceded the onset of MS. Among individuals who were initially EBV-seronegative, all patients who subsequently developed MS seroconverted before disease onset, and EBV infection was associated with an approximately 32-fold increased risk of developing MS. Furthermore, serum neurofilament light chain (NfL), a biomarker of neuroaxonal injury, increased only after EBV seroconversion, suggesting that neuronal damage follows, rather than precedes, EBV infection. Importantly, no comparable association was observed for any other human virus, providing the strongest epidemiological evidence to date supporting a central role for EBV in MS pathogenesis [39]. The principal longitudinal studies that have shaped our current understanding of the temporal association between EBV infection and MS are summarized in Table 1.

Overall, the epidemiological evidence linking EBV infection to MS is exceptionally strong; nevertheless, epidemiological studies alone cannot establish the precise biological mechanisms through which EBV contributes to disease development. Furthermore, although the association has been reproduced across diverse populations and study designs, epidemiological findings should be interpreted alongside mechanistic and experimental evidence to better understand how EBV may participate in MS pathogenesis.

## 4. EBV Genetic Variability and Its Clinical Implications in MS

While the epidemiological link between EBV and MS is well-established, investigations into whether naturally occurring sequence variation within the EBV genome—including polymorphisms and allelic variants in latent viral genes—confer increased disease risk have yielded complex and often contrasting results. Early investigations of EBV sequence diversity identified extensive polymorphisms within viral genes encoding immunogenic proteins. For example, sequencing of the EBNA1 and BRRF2 genes in EBV isolates from MS patients and controls revealed multiple sequence polymorphisms, with several variants occurring at marginally different frequencies in EBV strains infecting MS patients versus controls, suggesting that specific viral haplotypes might contribute to MS risk [46]. However, subsequent studies examining sequence variation in EBNA1 and the latent membrane protein LMP1 did not identify significant associations between these variants and MS susceptibility, indicating that not all EBV polymorphisms influence disease development [47]. More recent work has focused on highly variable regions of the EBV genome, including the EBNA2 gene, where specific allelic variants have been reported to occur more frequently in individuals with MS compared with healthy controls [48]. In addition, analyses of several polymorphic latent EBV genes have identified viral alleles that are enriched in MS patients or, conversely, more common in healthy individuals, suggesting that EBV strain diversity may modulate susceptibility to MS [49]. Nevertheless, findings across studies remain inconsistent, and further research is required to clarify the role of EBV genetic variation in MS pathogenesis.

Beyond sequence variation, integrative genomic analyses have demonstrated that the EBV transcription factor EBNA2 preferentially binds to genomic regions associated with MS susceptibility, including genes involved in immune signaling pathways such as CD40 [50], suggesting that viral and host genetic factors may converge in MS pathogenesis. In addition, emerging evidence indicates that EBV genetic variability may also influence therapy responses. For instance, patients with MS infected with different EBNA2 allelic variants show differential responses to pegylated interferon-β therapy, with individuals carrying the non-risk EBNA2 1.3B allele exhibiting enhanced antiviral responses and improved long-term clinical outcomes [51]. Together, these findings suggest that EBV genetic diversity may contribute not only to MS susceptibility but also to variability in host–virus interactions and treatment responses, highlighting the potential importance of viral genotyping in understanding disease mechanisms and guiding personalized therapeutic strategies.

In addition to sequence variation within protein-coding genes, EBV expresses several classes of non-coding RNAs, including BART-derived microRNAs (miR-BARTs), which contribute to the regulation of viral latency, immune evasion, and host immune responses. Although the role of EBV-derived microRNAs in MS has only recently begun to be explored, emerging evidence suggests that they may contribute to immune dysregulation associated with disease pathogenesis. Specifically, increased circulating levels of miR-BART17-5p, miR-BART10-5p, and miR-BART5-3p have been reported in patients with MS [52]. In addition, elevated expression of the EBV-derived microRNAs miR-BHRF1-2-5p and miR-BHRF1-3 has been detected in the circulation of MS patients and shown to correlate positively with Expanded Disability Status Scale (EDSS) scores, suggesting a potential association with disease severity [53]. However, direct evidence implicating EBV BART long non-coding RNAs (BART lncRNAs) in MS is currently lacking. Further studies are therefore required to determine whether these viral non-coding RNAs contribute to MS pathogenesis and whether they may serve as biomarkers or therapeutic targets.

## 5. EBV-Specific Antibody Responses in MS

Studies investigating EBV-specific antibodies have provided important insights into the relationship between EBV infection and MS. Patients with MS consistently exhibit higher antibody titers against EBV antigens, particularly EBNA1, compared with EBV-seropositive healthy controls. Anti-EBNA1 antibody levels are reported to be several-fold higher in different MS types and are associated with an increased risk of developing the disease [34,35,41,54]. Elevated EBNA1-specific antibody responses can already be detected during the prodromal phase before the onset of clinical symptoms [39,41,55], and antibody titers may continue to increase after disease onset and during relapses [25,41,56,57]. In addition to serum responses, EBV-specific antibodies, including antibodies against EBNA1, have also been detected in the cerebrospinal fluid (CSF) of patients with MS, indicating intrathecal humoral immune activity [58,59,60,61]. Increased antibody responses in MS are not restricted to EBNA1, as antibodies against several other EBV antigens—including viral capsid antigen (VCA), early antigen diffuse (EA-D), EBNA2, and EBNA3C—have also been reported to be elevated [43,62]. The elevated EBNA1-specific antibody responses have been attributed, at least in part, to genetic susceptibility factors, particularly the HLA-DRB1*1501 allele [63,64,65], the most well-known genetic risk factor of MS. Taken together, these findings indicate that EBV-specific antibody responses are consistently increased in MS and may precede clinical disease, although the functional significance of these elevated antibody titers remains unclear.

## 6. Impaired EBV-Specific Cell-Mediated Immune Responses in MS

**CD8^+^ and CD4^+^ T cells:** Cytotoxic T-cells (CD8^+^ T-cells) are essential for controlling EBV by identifying and killing virus-infected B cells, particularly through clonal expansion during acute IM. Ineffective control of EBV would therefore be expected to influence MS onset and/or progression. Evidence for dysregulation of EBV-specific T-cell responses in MS has been reported in several studies, although findings have not been entirely consistent. Some investigations have described increased CD8^+^ T-cell responses against EBV antigens in patients with MS [60], including elevated responses to latent EBV proteins and a higher frequency of CD8^+^ T-cells recognizing EBV epitopes compared with controls [66]. Similarly, increased activation of EBV-specific CD8^+^ T-cells has been observed particularly early in the course of MS, supporting a potential link between EBV-specific cellular immunity and disease onset [66]. Other studies have reported dynamic changes in EBV-specific CD8^+^ T-cell responses depending on disease activity, with expansion of CD8^+^ T-cells recognizing EBV lytic antigens during active disease [67]. EBV-expanded polyclonal T-cell lines have also been shown to exhibit autoreactivity against candidate CNS autoantigens, suggesting that EBV-driven T-cell responses may have the capacity to damage the CNS [68]. In contrast, additional studies have reported reduced EBV-specific cellular responses, including decreased CD8^+^ T-cell responses to EBV lytic antigens at disease onset and throughout later stages of MS, while CD8^+^ T-cells recognizing EBV latent antigens were increased but displayed reduced cytokine polyfunctionality consistent with T-cell exhaustion [69]. Furthermore, reductions in EBV-specific CD8^+^ T-cell responses have been observed during prospective follow-up of patients with clinically isolated syndrome, while EBV-specific CD4^+^ T-cell responses remained unchanged [70]. In addition to CD8^+^ T-cell responses, EBV-specific CD4^+^ T-cell responses have also been reported to be altered in the peripheral blood of patients with MS. Studies examining T-cell responses against the EBNA1 have demonstrated increased frequencies of EBNA1-specific CD4^+^ memory T-cells in MS patients, which exhibit enhanced proliferative capacity and increased interferon-γ production compared with healthy EBV carriers [71]. This enhanced response has been attributed to an expanded reservoir of EBNA1-specific central memory CD4^+^ T helper 1 precursors and Th1-polarized effector memory cells, indicating a selective increase in EBNA1-directed CD4^+^ T-cell immunity in MS [72].

Evidence has also emerged indicating that EBV-specific T-cell responses are present within the CSF of patients with MS. Analyses of T-cell receptor repertoires and antigen-specific T-cell responses have demonstrated intrathecal enrichment of EBV-reactive CD8^+^ T-cells in MS patients, suggesting a localized antiviral immune response within the central nervous system. Sequencing approaches revealed that EBV-reactive CD8^+^ T-cell receptor sequences, including public EBV-specific clonotypes, were selectively enriched in the CSF of MS patients and formed part of a clonally diverse yet compartmentalized and persistent intrathecal T-cell repertoire [73]. Similarly, highly expanded CD8^+^ T-cell clonotypes enriched in the CSF of MS patients were shown to recognize EBV antigens, and EBV-specific CD8^+^ T-cells were detected in patients whose CSF also contained EBV DNA and transcripts [74]. Additional studies have reported that a substantial proportion of expanded CSF T-cell clones are reactive to EBV-infected B cells, supporting the idea that EBV-specific T cells represent a major component of the intrathecal T-cell repertoire in MS [75]. Intrathecal enrichment of EBV-specific CD8^+^ cytotoxic T lymphocytes has also been observed in early MS, whereas such recruitment was not detected in other neurological diseases [76]. Although some analyses reported comparable frequencies of EBNA1-specific CD8^+^ T-cells in the CSF of MS and other inflammatory neurological diseases, these cells were consistently more frequent in CSF than in blood [77]. In addition to CD8^+^ T-cells, EBV-reactive CD4^+^ T-cells have also been detected in the CSF, including CD4^+^ T-cells recognizing EBV-transformed autologous B cells and EBV-specific CD4^+^ T-cells enriched in CSF-derived T-cell lines from MS and clinically isolated syndrome patients [78,79]. Together, these findings indicate that EBV-specific T cells, particularly CD8^+^ T-cells but also CD4^+^ T-cells, can accumulate within the intrathecal compartment in MS, supporting the presence of a localized EBV-directed immune response in the CNS. Although the studies discussed above collectively indicate that EBV-specific cellular immune responses are altered in MS, the reported findings are not always consistent. This heterogeneity likely reflects the complexity of EBV-specific cellular immunity, as differences in the EBV antigens examined, the immune cell populations analyzed, and the experimental approaches employed, together with biological heterogeneity among patient cohorts, make direct comparisons between studies challenging [80,81]. Consequently, further standardized longitudinal studies are required to clarify the functional significance of EBV-specific cellular immune responses in MS. Consequently, further standardized longitudinal studies are required to clarify the functional significance of EBV-specific cellular immune responses in MS.

**NK cells:** Natural killer (NK) cells are important components of the antiviral immune response and may contribute to immune control of EBV. Consequently, they may also contribute to MS pathogenesis. NK cells expressing cytotoxic receptors such as NKG2C and NKG2D have been shown to eliminate autoreactive cells recognizing CNS antigens cross-reactive with EBV-derived epitopes, suggesting that NK-cell-mediated cytotoxicity may contribute to the removal of potentially pathogenic autoreactive lymphocytes [82]. Consistent with a role for NK cells in antiviral immunity, alterations in NK-cell populations have also been reported in patients with MS, including reduced frequencies of specific NK-cell subsets, although these changes were not associated with EBV [83]. Evidence from studies of primary EBV infection further supports the importance of NK cells in controlling EBV. During IM, early differentiated CD56^dim^ NKG2A^+^ NK cells expand and display enhanced proliferation and degranulation when exposed to EBV-infected B cells expressing lytic antigens, indicating that these cells may contribute to immune control of EBV infection [84]. Experimental models have similarly shown that depletion of NK cells results in loss of immune control over lytic EBV infection, leading to increased disease severity and enhanced lymphocyte expansion [85]. Together, these findings suggest that NK cells participate in the immune response against EBV and that alterations in NK-cell activity or composition may influence host responses to EBV in the context of MS.

## 7. Molecular Mimicry Between EBV and CNS Antigens

**Figure 2:**
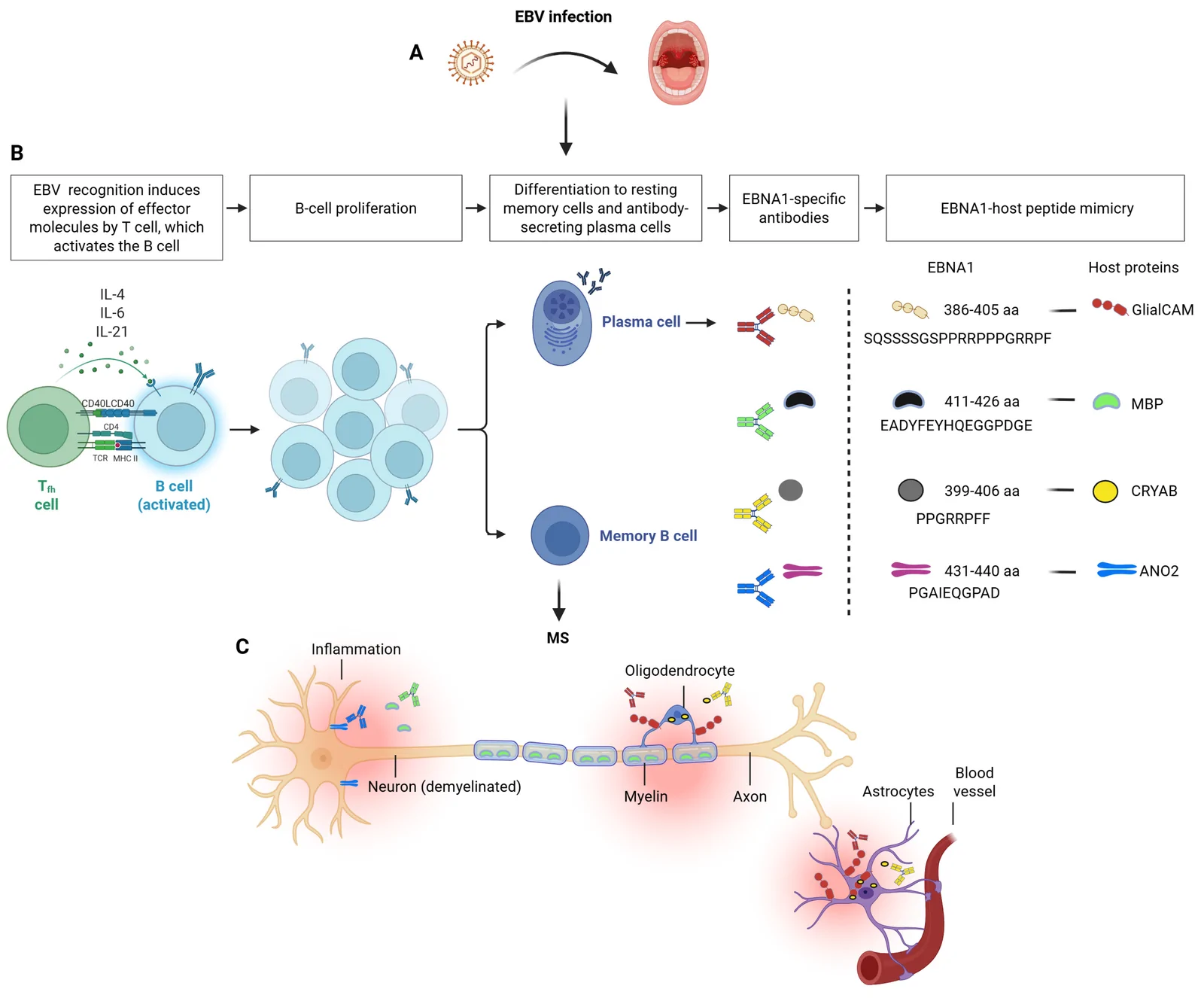
**EBNA1-mediated molecular mimicry as a proposed mechanism linking EBV infection to CNS autoimmunity. This figure illustrates a biologically plausible mechanism supported by experimental evidence; however, the precise contribution of molecular mimicry to MS pathogenesis remains incompletely understood.** (**A**) Following primary infection in the oropharynx, EBV infects B lymphocytes and establishes latency within the B-cell compartment. (**B**) Recognition of EBV antigens by CD4^+^ T helper cells leads to activation of EBV-infected B cells through costimulatory interactions and cytokine signaling. Activated B cells undergo clonal expansion and differentiate into plasma cells and memory B cells, generating antibodies directed against EBV antigens, including the latent viral protein EBNA1. Some EBNA1-specific antibodies can cross-react with structurally related host proteins expressed in the central nervous system through molecular mimicry. Identified EBNA1 peptide regions that exhibit sequence or structural similarity with host proteins include residues 386–405, 399–406, 411–426, and 431–440, which mimic epitopes present in GlialCAM, αB-crystallin (CRYAB), myelin basic protein (MBP), and anoctamin-2 (ANO2), respectively. (**C**) These cross-reactive antibodies may target CNS structures, including myelin and glial cells, potentially contributing to immune-mediated damage. If this mechanism occurs in vivo, the resulting inflammatory responses within the brain may promote demyelination of axons, activation of astrocytes and other glial cells, and disruption of neuronal integrity, hallmarks of multiple sclerosis pathology. Together, this model summarizes one of the leading proposed mechanisms by which EBV infection may initiate or amplify autoimmune responses against CNS antigens through EBNA1-mediated molecular mimicry and B-cell-driven immunity. While supported by substantial experimental evidence, the pathogenic significance of this mechanism and its contribution to disease initiation and progression remain areas of active investigation. ↓: progression to next phase.

One of the most widely proposed mechanisms linking EBV infection to MS is molecular mimicry, a process in which immune responses directed against viral antigens cross-react with structurally similar host proteins (Figure 2). EBV infection induces strong and persistent immune responses against several viral proteins, particularly the EBNA1, which is consistently expressed during latent infection and elicits robust antibody responses. Cross-reactive antibodies recognizing both EBNA1 and the glial cell adhesion molecule (GlialCAM) were identified from cerebrospinal fluid B cells of MS patients, with structural analyses demonstrating high-affinity molecular mimicry between the two antigens [59]. In addition, molecular mimicry was shown to be facilitated by a post-translational modification of GlialCAM, while EBNA1 immunization was shown to exacerbate disease in a mouse model of MS [59]. Subsequent studies have further supported this concept by showing elevated antibody responses against EBNA1 together with CNS proteins such as GlialCAM, alpha-crystallin B (CRYAB) [86,87], and anoctamin-2 (ANO2) in MS cohorts [87,88,89], with blocking experiments confirming cross-reactivity between EBNA1 and GlialCAM [87]. In the case of ANO2, reciprocal blocking experiments with ANO2 and EBNA1 peptides demonstrated antibody cross-reactivity, mapping to ANO2 [aa 140 to 149] and EBNA1 [aa 431 to 440] [89]. More recently, epitope-level analyses have further refined the molecular mimicry hypothesis by showing that immune responses directed against the EBNA1_381–452_ region cross-react with four distinct CNS-derived epitopes originating from GlialCAM_370–389_, CRYAB_2–21_, MBP_205–224_, and ANO2_135–154_ [90]. These findings indicate that EBNA1-directed antibodies may recognize multiple CNS antigens through shared epitopes, although further longitudinal studies are required to determine the contribution of these cross-reactive responses to MS pathogenesis.

In addition to antibody-mediated molecular mimicry, cross-reactive T-cell responses between viral and self-antigens have also been described in MS. Structural similarities between viral epitopes and host peptides can activate T-cells capable of recognizing both viral and self-antigens, providing a potential mechanism for autoimmune responses. Studies have shown that viral peptides can activate MBP-specific T-cell clones from MS patients, demonstrating that individual T-cell receptors can recognize structurally related peptides derived from EBV and MBP [91]. Consistent with this concept, cross-reactive CD8^+^ cytotoxic T-cells recognizing both MBP and peptides derived from EBV have been detected in MS, with increased precursor frequencies compared with controls. These T-cells were capable of directly inducing injury to oligodendrocytes expressing MBP and MHC class I molecules [92]. Cross-reactivity between EBV antigens and self-peptides has also been demonstrated for EBV-specific CD8^+^ T cells, which were shown to recognize both an immunogenic EBV epitope and a homologous human self-peptide presented by HLA-B*18:01 [93]. In addition to CD8^+^ T-cells, EBV-specific CD4^+^ T-cells may also exhibit cross-reactivity with CNS antigens, as expanded EBNA1-specific CD4^+^ T helper 1 cells in MS patients were reported to recognize myelin antigens more frequently than unrelated autoantigens [72]. Consistent with these findings, T-cell responses against the CNS antigen GlialCAM have also been reported in MS. Both CD4^+^ and CD8^+^ T-cells respond to GlialCAM, and CD4^+^ T-cells show enhanced interferon-γ production following stimulation with either EBNA1 or GlialCAM [87], indicating shared antigenic features. More recently, cross-reactive CD4^+^ T-cells recognizing both EBNA1 and the CNS antigen ANO2 have been identified, with overlapping T-cell receptor repertoires detected among EBNA1- and ANO2-specific T-cells isolated from MS patients [94]. Together, these observations indicate that, similar to antibody responses, EBV-specific T-cells can recognize structurally related CNS antigens, supporting a role for T-cell molecular mimicry in MS.

Collectively, the available evidence supports molecular mimicry as one of the most biologically plausible mechanisms linking EBV infection to MS. Nevertheless, several important questions remain unresolved. Although numerous cross-reactive antibody and T-cell responses between EBV antigens, particularly EBNA1, and CNS proteins have been identified, their precise contribution to disease pathogenesis, and whether these immune responses are sufficient to drive CNS pathology, remain uncertain. It is still unclear whether these immune responses represent an initiating event in CNS autoimmunity or whether they emerge as a consequence of ongoing inflammation and epitope spreading [6,95]. Furthermore, the factors that make EBNA1 the predominant target of cross-reactive immune responses, despite possessing multiple immune evasion, are not completely understood [6]. Addressing these questions will be essential for defining the pathogenic significance of molecular mimicry in MS and for guiding the development of safe EBV-targeted therapeutic and vaccine strategies.

## 8. EBV in the MS Brain: Evidence, Controversies, and Emerging Mechanisms

Investigations examining the presence of EBV in the brains of patients with MS have produced conflicting results. While several studies have reported detection of EBV in MS brain tissue [96,97,98,99,100,101,102,103], others using different experimental approaches have found little or no evidence of viral infection in the MS brain [104,105,106]. These discrepancies have been attributed in part to methodological differences and challenges associated with detecting EBV in pathological tissues, as well as variations in sample preparation and quality, as highlighted in a workshop review of the field [107]. In studies reporting EBV detection, analyses of post-mortem MS brain tissue have identified EBV infection in a substantial proportion of brain-infiltrating B cells and plasma cells [102]. Evidence for viral nucleic acids has been obtained through detection of EBV-encoded RNA (EBER) in MS brain lesions [96,100,101], as well as the identification of EBV latent and lytic gene transcripts in MS brain tissue [103]. In addition, EBV proteins have also been detected in the MS brain, including the latent proteins such as EBNA1, LMP1 and LMP2A [99,100,101], as well as lytic protein BZLF1 [100,101]. The conflicting results reported across pathological studies likely reflect not only biological heterogeneity among MS lesions, but also important methodological differences. Different studies have employed distinct approaches to detect EBV, including PCR-based assays, EBER in situ hybridization, immunohistochemistry, and more recently, spatial transcriptomic analyses, each targeting different viral nucleic acids or proteins and differing in analytical sensitivity and specificity [108]. Furthermore, accumulating evidence suggests that, when present, EBV-positive cells may be rare and spatially restricted within inflammatory infiltrates, making viral detection highly dependent on both the methodology employed and the tissue regions examined [107]. These considerations suggest that discrepant findings do not necessarily exclude a role for EBV in the MS brain but rather underscore the technical difficulty of demonstrating low-level or spatially restricted viral persistence in pathological CNS tissue. Accordingly, the low abundance of viral material, the marked heterogeneity of MS lesions, and the lack of universal reproducibility across independent laboratories remain important limitations when interpreting the available pathological evidence. Beyond detection studies, recent investigations have begun to explore how EBV-infected cells might contribute to CNS inflammation.

Spatial imaging analyses of MS brain tissue have identified EBV markers such as EBNA1 and LMP1 within lesions and described interactions between EBV-positive cells and glial and neuronal populations together with altered immune cell interactions and disruption of blood–brain barrier integrity [99]. In addition, transcriptomic studies have shown that EBNA2 and EBNA2-associated regulatory interactions affecting the CD40 pathway are detectable in MS brain tissue, suggesting that viral and host genetic factors may converge in MS-related pathogenic pathways [50]. Equally important, immunohistochemical evidence suggests that EBV-infected B cells further promote disease by utilizing the PD-1/PD-L1 checkpoint to evade T-cell mediated clearance in the brain [109]. This immune-evasion model is consistent with the aforementioned spatial studies indicating that EBV may exert effects that extend beyond the simple presence of infected cells within lesions. This high-dimensional analysis of MS brain tissue has suggested that EBV-positive cells are embedded within a broader disease-specific immune microenvironment characterized by altered cellular interactions and local tissue remodeling. In this context, PD-L1 expression by EBV-infected B cells, together with PD-1 expression on infiltrating T cells, may help sustain chronic inflammation by limiting effective antiviral clearance while preserving a compartmentalized immune infiltrate within the CNS. Although these recent spatial transcriptomic and imaging studies provide important new insights into the potential role of EBV within the MS brain, these observations remain preliminary and require independent validation in larger, well-characterized patient cohorts using standardized methodologies. Consequently, while these studies provide biologically plausible mechanistic insights, their reproducibility and pathological significance have yet to be firmly established.

In vivo experimental studies further support a role for EBV-associated B-cell responses in CNS inflammation. In humanized mouse models, EBV infection generates B-cell populations capable of homing to submeningeal brain regions and recruiting T-cells to the CNS [110]. Similarly, research in murine models suggests that while B cells naturally infiltrate the CNS during various infections, those that capture myelin antigens typically undergo rapid apoptosis in the absence of T-cell help. However, the EBV protein LMP1 can bypass this critical immunological checkpoint, providing a survival signal that enables myelin-reactive B cells to persist and drive inflammatory demyelination—a mechanism supported by the detection of LMP1 in the brains of MS patients [111].

**Figure 3:**
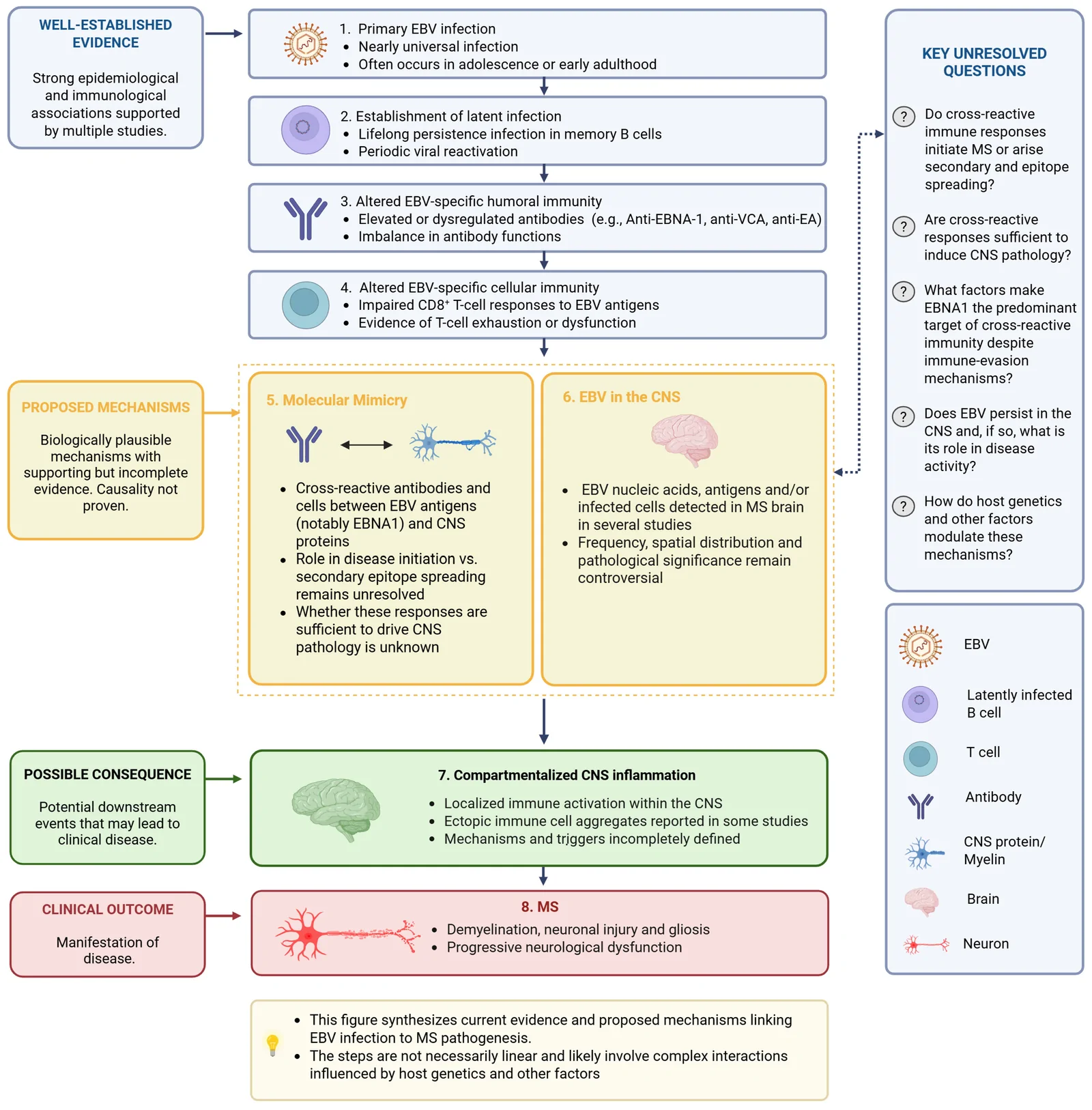
**Proposed integrated model linking EBV infection to MS pathogenesis.** Strong epidemiological evidence indicates that EBV infection precedes the development of MS, although the precise causal mechanisms remain incompletely understood. Following primary infection and lifelong latency in B lymphocytes, EBV may contribute to MS through multiple, potentially interconnected mechanisms, including dysregulated humoral immunity, altered EBV-specific cellular immune responses, molecular mimicry between EBV and CNS antigens, and the persistence of EBV-infected immune cells within the CNS. Together, these processes may promote compartmentalized CNS inflammation, immune dysregulation, and demyelination, ultimately contributing to MS pathology. This schematic represents a proposed integrative model based on the current evidence discussed throughout this review and should not be interpreted as a definitive pathogenic pathway. The relative contribution of each mechanism and their interactions remain to be fully elucidated.

Taken together, the available evidence provides biologically plausible mechanisms linking EBV to CNS inflammation in MS. However, the frequency, distribution, and pathological significance of EBV within the MS brain remain controversial. Future studies integrating standardized pathological approaches with emerging spatial technologies, together with independent validation across laboratories, will be essential to determine whether and under which pathological conditions EBV contributes directly to compartmentalized CNS inflammation in MS. Although each of the proposed mechanisms is supported by varying degrees of experimental and clinical evidence, they are unlikely to operate in isolation. Rather, interactions between dysregulated antiviral immunity, molecular mimicry, and EBV persistence may collectively contribute to CNS inflammation and disease pathogenesis. A schematic overview integrating these proposed mechanisms and highlighting the key unresolved questions is presented in Figure 3.

## 9. Future Directions and Outstanding Questions

The substantial body of epidemiological, immunological, and experimental evidence linking EBV infection to MS strongly suggests that the virus may play an important role in disease initiation and/or progression. These observations highlight the potential value of strategies aimed at preventing EBV infection or enhancing antiviral immune control, including the development of prophylactic EBV vaccines and immunotherapies targeting EBV-infected cells. Support for the therapeutic relevance of EBV-directed immunity in MS comes from a limited number of studies investigating adoptive transfer of EBV-specific T-cells. In pioneering work by Pender and colleagues, adoptive EBV-specific T-cell therapy targeting the viral antigens EBNA1, LMP1, and LMP2 was associated with neurological improvement in patients with progressive MS, including reductions in the number of brain lesions and intrathecal IgG production [112]. Subsequently, an open-label phase I study reported symptomatic improvement in MS patients receiving EBV-specific T-cells, including reduced fatigue and, in some cases, improvement in Expanded Disability Status Scale (EDSS) scores [113]. Importantly, long-term follow-up of these patients revealed that clinical benefits were sustained in a subset of individuals for up to 2–3 years after treatment, with persistent improvements in fatigue, disability scores, and other neurological symptoms, while no serious treatment-related adverse events were observed [114]. Notably, sustained clinical improvement correlated with higher EBV-specific CD8^+^ T-cell reactivity in the administered T-cell product, suggesting that therapeutic efficacy depends on the functional potency of the infused cells. These clinical observations may reflect enhanced EBV-specific polyfunctional T-cell responses capable of eliminating EBV-infected B cells, which have been proposed to contribute to MS pathogenesis.

The development of a prophylactic EBV vaccine, which is currently lacking, could theoretically reduce the risk of MS. In uninfected individuals, particularly children and adolescents, such a vaccine could potentially provide sterile immunity against EBV infection. Even in the absence of sterile immunity, vaccination might reduce the incidence of IM, which has been consistently associated with an increased risk of MS [115]. Over the past decades, several EBV vaccine strategies have been explored, targeting different viral antigens including gp350, gH/gL, gp42, EBNA1, EBNA3A, and LMP1 [116]. These approaches, based on multiple vaccine platforms, have demonstrated encouraging safety profiles and the ability to induce EBV-specific immune responses in early studies. However, a deeper characterization of the quality and specificity of vaccine-induced immune responses will be essential before prophylactic or therapeutic EBV vaccines can be broadly implemented. This is particularly important given the complexity of EBV-specific humoral and cellular immune responses implicated in MS pathogenesis. Indeed, the optimal antigen composition of a prophylactic vaccine may differ from that of a therapeutic vaccine intended for individuals with established MS. For example, inclusion of EBV antigens or epitopes involved in molecular mimicry with central nervous system proteins could theoretically exacerbate autoimmune responses rather than prevent them, particularly if sterile immunity against EBV infection cannot be achieved. Careful antigen selection will therefore be critical in the design of EBV vaccines aimed at reducing MS risk or modifying disease progression. Until such vaccine strategies become available, the exploration of anti-EBV therapies may represent a more immediately achievable approach. Existing therapies that broadly target B cells are already used in MS and have demonstrated significant clinical benefit [117,118]. However, the development of treatments specifically directed against EBV and/or EBV-infected cells could provide a more targeted strategy while minimizing unintended effects on immune cells responsible for protection against other pathogens. Recent data suggest that this approach may be feasible, as monoclonal antibodies targeting EBV glycoproteins such as gp350 and gp42 have shown strong virus-neutralizing activity [119]. In parallel, growing interest has focused on the repurposing of licensed drugs with anti-EBV activity as a practical therapeutic strategy for MS. Since de novo drug development is costly and time-consuming, repurposing existing agents offers a faster route to clinical translation. A recent systematic evaluation identified several licensed compounds, including famciclovir, maribavir, spironolactone, and tenofovir alafenamide, as promising candidates based on their anti-EBV activity, safety, tolerability, and potential suitability for clinical trials in MS [120]. These agents primarily target lytic EBV replication and may help reduce viral reactivation or EBV-driven immune activation. Importantly, emerging evidence suggests that effective EBV-targeted therapy in MS may need to address both the lytic and latent phases of the viral life cycle, as latent–lytic cycling within EBV-infected B cells may contribute to persistent immune activation and chronic neuroinflammation. In this context, novel therapeutic approaches targeting latent viral proteins, particularly EBNA1 [121], are of considerable interest, as inhibition of EBNA1 could disrupt maintenance of latent EBV episomes and promote selective elimination of persistently infected cells, thereby offering a more comprehensive strategy for targeting EBV-driven pathology in MS.

Although recent advances in EBV-targeted therapies are encouraging, their translation into clinical practice remains limited. Evidence supporting adoptive EBV-specific T-cell therapy is currently derived from relatively small clinical studies, and larger randomized controlled trials are required to establish efficacy and long-term safety. Likewise, although conventional antiviral agents may reduce lytic EBV replication and viral reactivation, their impact on latent infection remains limited. Consequently, whether targeting lytic replication alone is sufficient to provide meaningful clinical benefit in MS remains uncertain, highlighting the need for therapeutic strategies capable of addressing both latent and lytic phases of the EBV life cycle.

Equally important, several key mechanistic aspects of EBV-associated autoimmunity in MS remain incompletely understood. In particular, further investigation is needed to clarify the role of molecular mimicry between EBV antigens and central nervous system (CNS) proteins. Although recent studies have identified several cross-reactive antibodies [59,86,87,90], future work should determine the prevalence of these antibodies across different MS subtypes and stages of disease. In addition, comprehensive characterization of the functional properties of these antibodies, including their affinity and avidity for both viral and self-antigens, will be important for assessing their potential pathogenic relevance. Such analyses may help determine whether cross-reactive immune responses represent a driving force in MS pathogenesis or instead reflect secondary immune activation. A deeper understanding of these mechanisms will also be essential for evaluating the potential implications of EBV-targeted vaccines and immunotherapies in the context of MS.

## 10. Conclusions

In summary, a growing body of epidemiological, immunological, and experimental evidence supports a central role for EBV in MS pathogenesis. Multiple mechanisms—including dysregulated antiviral immunity, molecular mimicry between EBV and CNS antigens, and persistence of EBV-infected B cells in the CNS—have been proposed to contribute to disease development. However, important questions remain regarding the relative contribution of these mechanisms and the conditions under which EBV infection leads to autoimmune pathology. In our view, future research should move beyond establishing associations and focus on defining the pathogenic mechanisms linking EBV to MS. Particular emphasis should be placed on determining whether EBV-specific cross-reactive antibodies and T cells directly alter the function of CNS-resident cells and on investigating whether qualitative features of the anti-EBNA1 immune response, such as antibody avidity and functional properties, rather than antibody titers alone, distinguish patients with MS from EBV-seropositive healthy individuals. Furthermore, these mechanisms should be investigated across the different clinical phenotypes of MS, as relapsing–remitting, secondary progressive, and primary progressive disease may differ in their dependence on EBV-driven immune responses. Addressing these questions will be essential for clarifying the pathogenic role of EBV and for identifying the patient populations most likely to benefit from future EBV-targeted preventive and therapeutic strategies.

## Figures and Tables

**Table 1:** Key longitudinal studies investigating the association between EBV and MS.

Study	Study Population/Design	Key Finding	Contribution to EBV–MS Relationship
[40]	Historical prospective Danish cohort	IM was associated with a 2.8-fold increased risk of MS, with MS developing only after IM.	First prospective evidence linking symptomatic primary EBV infection with increased MS risk.
[41]	Prospective nested case–control study (83 MS cases, 166 controls)	Anti-EBNA antibody titres increased years before the onset of clinical MS.	Demonstrated that elevated EBV-specific immune responses precede disease onset.
[42]	Pediatric MS cohort study	Nearly all children with MS were EBV-seropositive, with no evidence of recent primary infection.	Extended the temporal association between EBV infection and MS to paediatric-onset disease.
[31]	Prospective nested case–control study (305 MS cases, 610 controls)	All initially EBV-seronegative individuals who developed MS seroconverted before disease onset.	Established the temporal sequence between primary EBV infection and MS development.
[43]	Prospective validation cohort (222 MS cases, 444 controls)	High anti-EBNA antibody titres were associated with a markedly increased risk of MS.	Confirmed anti-EBNA antibodies as robust preclinical biomarkers of MS risk.
[44]	Nurses’ Health Study prospective cohort	Higher EBV-neutralizing antibody titres were not protective against MS after adjustment for anti-EBNA1 antibodies.	Suggested that neutralizing antibodies provide limited additional predictive value beyond anti-EBNA1 responses.
[45]	Population-based genetic association study	HLA-DR alleles combined with anti-EBV antibody profiles markedly improved MS risk prediction.	Suggested that HLA-associated susceptibility may be mediated, in part, through EBV-specific immune responses.
[39]	20-year prospective cohort of >10 million U.S. military personnel	EBV infection increased MS risk ~32-fold; EBV seroconversion preceded both MS onset and neurofilament light chain elevation; no other virus showed a comparable association.	Provided the strongest longitudinal evidence supporting EBV as a necessary antecedent of MS.

## Data Availability

No new data were created or analyzed in this study. Data sharing is not applicable to this article.

## Abbreviations

The following abbreviations are used in this manuscript:

EBV	Epstein–Barr virus
MS	Multiple sclerosis
CNS	Central nervous system
EBNA1	Epstein–Barr nuclear antigen 1
GlialCAM	Glial cell adhesion molecule
ANO2	Anoctamin 2
CRYAB	Alpha-B crystallin
MBP	Myelin basic protein
